# Comparative Effects of C3 and C4 Forages on Growth Performance, Digestibility, and Nitrogen Balance in Korean Crossbred Black Goats

**DOI:** 10.3390/ani15172569

**Published:** 2025-09-01

**Authors:** Xue-Cheng Jin, Seong-Jin Kim, Won-Young Lee, Hyun-Jung Park, Jeong-Sung Jung, Na-Yeon Kim

**Affiliations:** 1 Department of Animal Science, Konkuk University, Seoul 05029, Republic of Korea; kimhs@live.com; 2 Asia Pacific Ruminant Institute, Icheon 17385, Republic of Korea; apri76@daum.net; 3 Department of Livestock, Korea National University of Agricultures and Fisheries, Jeonju 54874, Republic of Korea; eewy81@korea.kr; 4 Department of Animal Biotechnology, Sangji University, Wonju 26339, Republic of Korea; parkhj02@sangji.ac.kr; 5 Grassland and Forages Division, Department of Animal Resources Development, National Institute of Animal Science, Chonan 31000, Republic of Korea; jjs3873@korea.kr

**Keywords:** digestibility, forages, growth performance, Korean crossbred black goats, nitrogen utilization

## Abstract

This study compared the effects of two C3 forages (Italian ryegrass [RG], Timothy grass [TG]) and two C4 forages (Klein grass [KG], Bermuda grass [BG]) on growth performance, nutrient digestibility, and nitrogen (N) balance in Korean crossbred black goats to evaluate C4 warm-season forages as alternatives under changing climate conditions. Sixteen castrated goats (10 months old) were allocated to four treatments using a randomized complete block design. Diets contained 40% treatment-specific forage and 60% commercial concentrate. After adaptation periods, a 5-day metabolism trial measured performance and digestibility parameters. No significant differences occurred among treatments for growth performance or digestibility of dry matter, crude protein, neutral detergent fiber, ash, and non-fiber carbohydrate. C4 grasses showed significantly lower acid detergent fiber and ether extract digestibility than C3 grasses, with KG having the lowest ether extract digestibility. The KG group had higher N intake and absorption than the TG group, while BG showed lower urinary and total N excretion than KG. No differences existed in retained N, utilization efficiency, or biological value among groups. Both C3 and C4 forages supported comparable goat growth performance, providing a reference for utilizing different photosynthetic pathway forages under changing climatic conditions.

## Simple Summary

Climate change is creating challenges for traditional cool-season grass production. This study aimed to evaluate whether warm-season grasses could effectively replace cool-season grasses in goat diets without compromising animal performance. Sixteen young Korean crossbred black goats were fed diets containing either cool-season grasses (Italian ryegrass or Timothy grass) or warm-season grasses (Klein grass or Bermuda grass) combined with commercial concentrate feed. Researchers measured growth performance, nutrient digestibility, and nitrogen utilization over a controlled feeding trial. Results demonstrated that goats fed warm-season grasses achieved comparable growth performance to those fed traditional cool-season grasses. While differences occurred in fat and fiber digestibility between grass types, overall nutrient utilization remained satisfactory across all treatments. These findings indicate that warm-season grasses can serve as viable alternatives to cool-season forages in goat production systems.

## 1. Introduction

Climate change is one of the most pressing challenges facing global agriculture systems [1]. In South Korea, a temperate climate country, warming trends are particularly evident. According to the Korea Meteorological Administration, the average temperature has risen by 1.4 degrees Celsius over the past 30 years, with a notable rise in summer heatwave days [2,3]. This climate shift has significant implications for forage production systems that traditionally relied on C3 cool-season grasses adapted to temperate conditions [4]. Specifically, these grasses now face growth limitations during extended hot periods, resulting in reduced yields and declining nutritional quality [5]. These limitations potentially compromise the ability to meet livestock feed demands throughout the year [6]. As these warming trends continue, there is emerging potential for introducing C4 warm-season forages that may better withstand these changing conditions [7]. However, the inherent physiological and biochemical differences between C3 and C4 plants indicate that this transition faces certain challenges [8].

C3 and C4 plant groups are distinguished by their initial carbon fixation pathways during photosynthesis, which determine both their environmental adaptability and nutritional characteristics [9]. C3 plants produce a 3-carbon molecule (3-phosphoglycerate) as the first stable product of photosynthesis, catalyzed by Rubisco, combining RuBP with CO_2_ [10]. These plants typically exhibit higher overall nutritional quality under moderate temperatures (10–25 °C), and are thus widely used as traditional forage resources in temperate regions [11,12]. In contrast, C4 plants employ a more complex carbon fixation mechanism, generating a 4-carbon molecule (such as oxaloacetate) as their initial product [13]. This process is catalyzed by phosphoenolpyruvate (PEP) carboxylase, combining PEP with bicarbonate (HCO_3_^−^) rather than directly with CO_2_, creating an additional carbon-concentrating mechanism [10]. This biochemical adaptation allows C4 plants to photosynthesize more efficiently at higher temperatures (25–35 °C) and under water-limited conditions, making them potentially valuable forage resources in warming climates [14,15]. However, compared to C3 plants, C4 forages typically contain more structural carbohydrates and lignin, which may be due to accelerated cell wall lignification under heat stress [16,17]. This elevated fiber content typically reduces ruminal digestibility, thereby potentially compromising the nutritional value of C4 forages for ruminants despite their superior environmental resilience under warming conditions [18]. Therefore, it is necessary to investigate the specific effects of these different forage types on ruminant digestibility.

Several studies have investigated the digestibility of C3 and C4 forages in small ruminants. Robinson et al. [19] found no differences in dry matter (DM) digestibility between C3 grass (Tall fescue) and C4 grass (Bermuda grass) in Boer goats, though their investigation did not fully characterize fiber digestibility parameters. Similarly, Archimède et al. [20] observed no overall DM digestibility differences between C4 and C3 grasses, but unexpectedly reported higher neutral detergent fiber (NDF) and acid detergent fiber (ADF) digestibility in C4 grasses. Notably, the study revealed that C4 grasses exhibited enhanced digestibility in tropical environments compared with temperate regions, suggesting the presence of significant environment-animal adaptation interactions. These contradictory findings underscore the necessity to investigate digestibility responses of locally adapted ruminants to different forage types under specific regional conditions. Especially in transitional climate zones like Korea, both C3 and C4 forage types may become increasingly important. However, comparative nutritional studies of C3 and C4 forages for goats under these conditions are still limited.

Therefore, we hypothesize that C4 warm-season forages may demonstrate nutritional value and production effects comparable to traditional C3 cool-season forages for goats. This study aimed to investigate the effects of two C3 forages (Italian ryegrass [RG], *Lolium multiflorum*; Timothy grass [TG], *Phleum pratense*) and two C4 forages (Klein grass [KG], *Panicum coloratum*; Bermuda grass [BG], *Cynodon dactylon*) on growth performance, apparent nutrient digestibility, and nitrogen (N) balance in Korean crossbred black goats.

## 2. Materials and Methods

### 2.1. Animal Ethics Statement

All animal experiments strictly adhered to the guidelines provided by the Institutional Animal Care and Use Committee (IACUC) of Sangji University (approval protocol #2024-6).

### 2.2. Animals, Experimental Design, and Diets

**Figure 1:**
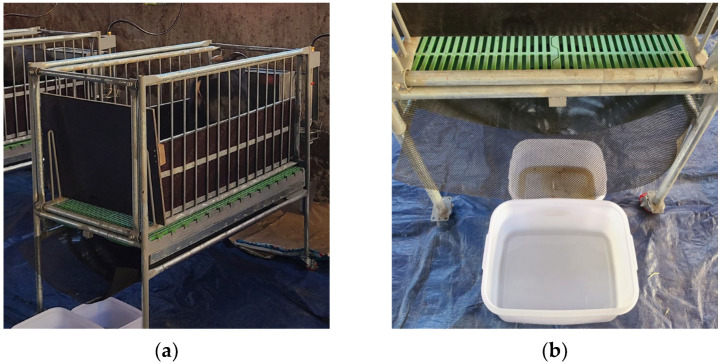
Metabolic cage system used for nutrient digestibility and nitrogen balance measurements. (**a**) Overview of individual metabolic cage showing the elevated housing unit with slatted flooring for animal confinement; (**b**) Detailed view of the separation and collection system beneath the cage for precise separation and quantitative collection of feces and urine.

Sixteen castrated Korean crossbred black goats (Korean native black × Boer, 10 months old, initial body weight [BW]: 43.3 ± 2.4 kg) were used in this study. A randomized complete block design was employed, with goats divided into four sequential blocks based on body weight (four goats per block). Within each block, goats were randomly assigned to one of four treatment groups: RG (C3 plant), TG (C3 plant), KG (C4 plant), and BG (C4 plant). The experimental diets consisted of 40% forage (treatment-specific grass type) and 60% commercial concentrate (Farmsco Inc., Anseong, Republic of Korea). These components were thoroughly mixed to meet NRC (2007) [21] nutritional requirements for goats. Animals were fed twice daily at 07:00 and 17:00 h, with ad libitum access to feed and water throughout the experimental period. Prior to the experimental period, all goats were adapted to the experimental diets for 7 days in individual pens (1.5 m long × 1.5 m wide). Subsequently, goats were transferred to individual metabolic cages (2 m long × 1.0 m wide), where a 2-day adaptation period to the caging environment was allowed. The metabolic cages were specifically designed to allow precise separation and collection of feces and urine (Figure 1).

The experimental period lasted 5 days, during which feed refusals, feces, and urine were collected daily before morning feeding. Daily health checks were performed throughout the experiment. Body weights were measured before morning feeding at the beginning and end of the experiment, and average daily gain (ADG) and feed efficiency (gain/feed as DM) were calculated.

### 2.3. Chemical Composition Analysis

The nutritional composition of the experimental diets was analyzed according to standardized procedures established by the AOAC (1990) [22]. Determination of DM content was accomplished through overnight drying of ground feed samples in a vacuum oven maintained at 100 °C (method 930.15). For crude protein (CP) analysis, a Kjeldahl analyzer (Kjeltec 2300, FOSS Analytical, Hilleroed, Denmark) was employed to measure total N content, which was subsequently converted to CP using the factor 6.25 (method 984.13). Ether extract (EE) content was quantified utilizing an extraction system (ANKOM XT15 Extractor, ANKOM Technology, Macedon, NY, USA) following method 920.39. Ash content was assessed by overnight incineration of samples at 550 °C in a muffle furnace (KMF-500, Lab Corporation, Seoul, Republic of Korea) in accordance with method 942.05. The NDF and ADF were evaluated following the analytical protocols described by Van Soest et al. [23]. The nutritional components of the experimental forages and commercial concentrate are shown in Table 1.

Daily fecal samples were thoroughly homogenized, and a representative subsample (approximately 10% by weight) was collected. These daily subsamples were composited by animal over the collection period to create a single composite sample per animal. Composite fecal samples were dried in a forced-air oven at 60 °C for 72 h to determine DM content and then ground to pass through a 1-mm screen. The dried fecal samples were analyzed for CP, EE, ash, NDF, and ADF following the same analytical procedures used for the feed samples. Total urine output was collected daily in containers with 50 mL of 6 N HCl to prevent N volatilization. A representative subsample (10% of daily urine volume) was collected, and these daily subsamples were composited by animal over the collection period. The composite urine samples were stored at 4 °C until analyzed for total N content using the same Kjeldahl method as for feed samples.

### 2.4. Digestibility and Nitrogen Balance Calculations

Daily dry matter intake (DMI) was calculated by subtracting the dry weight of feed refusals from the dry weight of feed offered. Daily nutrient intake was calculated by multiplying the DMI by the concentration of each nutrient in the diet. The apparent digestibility coefficients for DM, CP, EE, NDF, and ADF were calculated using the following equation: Apparent digestibility (%) = [(Nutrient intake − Fecal nutrient output) ÷ Nutrient intake] × 100.

For N metabolism assessment, daily excretion of fecal nitrogen (FN) was determined by multiplying the DM weight of daily fecal output by its N concentration, while urinary nitrogen (UN) excretion was calculated by multiplying the total daily urine volume by its N concentration. Nitrogen utilization was further characterized through several calculations: digestible nitrogen (DN, g/d) was calculated as nitrogen intake (NI) minus FN, with nitrogen digestibility (ND, %) determined as (DN/NI) × 100. Total excreted nitrogen (TN, g/d) was calculated as the sum of FN and UN, with nitrogen excretion percentage calculated as (TN/NI) × 100. Retained nitrogen (RN, g/d) was calculated as DN minus UN, with nitrogen retention efficiency (RN/NI, %) calculated as (RN/NI) × 100. Finally, the biological value of nitrogen (RN/DN, %) was determined as (RN/DN) × 100.

### 2.5. Statistical Analysis

All data were analyzed using the MIXED procedure in SAS software (version 9.4, SAS Institute Inc., Cary, NC, USA), with grass type as a fixed effect and experimental week as a random effect. Multiple comparisons between grass treatments were adjusted using the Tukey’s test. The initial BW was included as a covariate for growth performance. Additionally, to compare differences between photosynthetic pathway types, specific contrast tests were performed using the CONTRAST statement to compare C3 forages (RG and TG) with C4 forages (KG and BG). Each grass type was assigned equal weight in the tests. Statistical significance was defined as *p* < 0.05. Values where 0.05 ≤ *p* < 0.1 were considered to indicate a trend.

## 3. Results and Discussion

### 3.1. Growth Performance of Goats Fed C3 and C4 Forages

This study investigated the effects of two C3 forages (RG and TG) and two C4 forages (KG and BG) on growth performance, apparent nutrient digestibility, and nitrogen balance in Korean crossbred black goats. Growth performance results indicated no significant differences (*p* > 0.05) among goats fed different forage types, regardless of their photosynthetic pathway (Table 2).

This observation contradicts the conventional understanding that C3 grasses typically support better animal performance than C4 grasses due to their higher nutritional quality [16]. This may be primarily attributed to the high proportion of concentrate supplementation (60% of the diet) included in the experimental diet to meet the nutritional requirements of goats, which may have masked potential differences in forage quality.

Although not statistically significant (*p* = 0.07), the numerically higher ADG observed in goats fed BG (0.4 kg/d compared to approximately 0.1 kg/d for other treatments) warrants attention, particularly considering that BG had the highest NDF (73.6%) and lowest NFC (9.0%) among all forages tested. This unexpected performance pattern highlights that conventional nutritional metrics may not fully predict growth responses in goats, particularly when high levels of concentrate are included in the diet. Future research with a larger sample size over longer periods of time, combined with investigating rumen fermentation characteristics, may reveal more definitive patterns. Furthermore, the similar DMI across treatments indicates that palatability was not a limiting factor for C4 forages. Therefore, despite the biochemical and structural differences between C3 and C4 plants, both feed types supported similar growth performance in Korean crossbred black goats when incorporated at 40% of the diet.

### 3.2. Nutrient Digestibility Differences Between C3 and C4 Forages

C4 grasses typically contain higher proportions of structural carbohydrates and lignin, which reduce fiber digestibility [18]. Lee et al. [5] reported that fiber content increases by 13% for each 1 °C rise in temperature. Interestingly, the C4 forages used in this study exhibited lower ADF content (26.2% for KG, 27.4% for BG) compared to C3 grasses (33.8% for RG, 38.1% for TG), while their NDF content (64.6% for KG, 73.6% for BG) was slightly higher than that of C3 forages (61.3% for RG, 69.5% for TG). These variations may be attributed to differences in maturity stage, cultivation environment, harvest season, and cultivar improvements of forage grasses [25,26]. Despite their unexpectedly lower ADF content, C4 forages still demonstrated significantly reduced ADF digestibility (*p* < 0.01), with the BG group showing particularly lower values compared to RG and TG groups (*p* < 0.05) (Table 3). This reduction may be attributed to the higher proportion of lignin in the ADF fraction of C4 grasses compared to C3 grasses [27]. As a structural factor, the Kranz anatomy characteristic of C4 plants, which leads to thickened bundle sheath cells and more rigid leaf texture, may contribute to decreased ADF digestibility [10].

Notably, NDF digestibility showed no significant differences among the goat groups (*p* > 0.05). This finding may be explained by the superior ability of goat ruminal bacteria to degrade hemicellulose/xylan components [28]. Additionally, Korean crossbred black goats may possess breed-specific ruminal characteristics that enhance fiber degradation [29,30]. However, the complex interactions between lignin content, lignin types, and the ruminal environment make direct interpretation challenging without more detailed analysis of fiber subcomponents and their digestion kinetics.

Apparent EE digestibility was significantly influenced by forage type (*p* < 0.01), with C3 grasses showing higher digestibility than C4 grasses overall (*p* = 0.01). Specifically, the EE digestibility of the KG group was significantly lower than that of the TG and RG groups (C3 forages), and even lower than that of BG, another C4 forage (*p* < 0.05). Meanwhile, the EE digestibility of the BG group was comparable to C3 grasses (*p* > 0.05). This suggests that the lower EE digestibility associated with C4 grasses is not a general characteristic of the C4 photosynthetic pathway itself, but rather a specific effect associated with KG. One possible explanation is that steroidal saponins in KG may form micelles with bile acids, thereby reducing the availability of bile acids to form micelles with fatty acids [31,32]. However, current research has not yet identified the saponin components in the forages, and direct evidence linking KG to reduced EE digestibility in goats still requires further investigation. Furthermore, consistent with multiple previous studies, no significant differences were observed in the digestibility of DM, CP, Ash, and NFC among various forage types (*p* > 0.05) [19,20,33]. This suggests that the bioavailability of these components is minimally influenced by forage type.

### 3.3. Nitrogen Balance and Utilization Efficiency

Compared to goats fed C3 grasses, those fed C4 grasses exhibited significantly higher NI (*p* = 0.02), DN (*p* < 0.01), and TN (*p* = 0.01) (Table 4). Within the C4 group, KG-fed goats demonstrated significantly higher NI and DN than TG-fed goats, while their TN was significantly higher than both TG and BG groups (*p* < 0.05). These differences directly reflect the CP content of the forages used in this study, where C4 grasses (11.1% for KG, 8.3% for BG) contained higher protein levels than C3 grasses (7.0% for RG, 7.8% for TG). Although this contradicts the common understanding that C3 plants typically have higher protein content than C4 plants, evidence suggests that varietal improvement may have overcome the traditional limitations imposed by photosynthetic pathways on protein accumulation [34].

Despite the differences in N throughput, RN showed no significant differences between any treatment groups or between C3 and C4 grasses (*p* > 0.05). These findings contrast with those reported by Robinson et al. [19], who observed that camels and goats retained more nitrogen from C4 forage. This discrepancy may be attributed to differences in the specific forage species compared across studies. Notably, within the C4 grass treatments, the KG group exhibited significantly higher UN excretion than the BG group (*p* < 0.05). This suggests that a substantial portion of the additional N consumed in the KG group was deaminated and excreted as urea rather than being incorporated into microbial protein or animal tissue. This suggests possible asynchrony between N release and ruminal energy availability, or a suboptimal amino acid profile relative to animal requirements [35].

Interestingly, ND, RN/NI, and RN/DN showed no significant differences across treatments (*p* > 0.05). This indicates that in this high-concentrate feeding system, the proportional efficiency of N digestion and the biological value of absorbed N were similar across different forage types. The concentrate feed likely provided a consistent baseline of energy, resulting in comparable efficiency of N utilization despite higher absolute N throughput for C4 grasses. Surprisingly, BG, a C4 grass, demonstrated excellent protein retention efficiency (RN/NI = 50.3%, highest though not significantly different) and biological value of absorbed N (RN/DN = 71.6%, second only to TG). This may explain its trend toward higher ADG, suggesting it could be a valuable protein source for ruminants. The differential responses in N metabolism between KG and BG (both C4 plants) highlight that species selection within photosynthetic groups is as important as comparisons between C3 and C4 classifications.

## 4. Conclusions

Despite some differences in specific digestibility parameters, both C3 and C4 forages can support comparable growth performance in Korean crossbred black goats when incorporated at 40% of the diet. The lower ADF digestibility in C4 forages was not sufficient to negatively impact overall animal performance, suggesting that goats possess adaptive mechanisms to effectively utilize diverse forage resources. These findings support the adoption of C4 warm-season forages as a sustainable feeding strategy in goat production systems. Future studies involving longer feeding periods and rumen microbiota analysis would enhance understanding of C3 versus C4 forage effects in goats.

## Figures and Tables

**Table 1:** Chemical composition of experimental forages and concentrate (% of DM).

Items	C3 Forages		C4 Forages		Concentrate
	RG	TG	KG	BG	
DM (% of as-fed)	88.3	91.6	91.9	85.9	89.1
CP	7.0	7.8	11.1	8.3	18.3
N	1.1	1.2	1.8	1.3	2.9
EE	2.1	2.3	3.3	1.5	4.6
NDF	61.3	69.5	64.6	73.6	31.4
ADF	33.8	38.1	26.2	27.4	11.7
Ash	7.1	6.3	8.5	7.5	8.3
NFC ^1^	22.5	14.2	12.5	9.0	37.5

^1^ Non-fiber carbohydrate (%) = 100 − (CP + EE + NDF + Ash) [24]. RG, Italian ryegrass; TG, timothy grass); KG, Klein grass; BG, Bermuda grass; DM, dry matter; CP, crude protein; N, nitrogen; EE, ether extract; NDF, neutral detergent fiber; ADF, acid detergent fiber; NFC, non-fiber carbohydrate.

**Table 2:** Growth performance of Korean crossbred black goats fed diets containing different forage types.

Items	C3 Forages		C4 Forages		SEM	*p*-Value	
	RG	TG	KG	BG		(Forage Type)	(C3/C4 Pathways)
Initial BW (kg)	44.9	43.7	43.3	43.5	0.62	0.253	0.126
Final BW (kg)	45.3	44.0	43.5	43.2	0.54	0.163	0.281
ADG (kg/d)	0.1	0.1	0.1	0.4	0.05	0.073	0.154
DMI (kg/d)	0.9	0.8	0.9	0.9	0.03	0.112	0.183
Feed efficiency (kg gain/kg of DM)	0.1	0.1	0.1	0.4	0.05	0.154	0.284

RG, Italian ryegrass; TG, timothy grass); KG, Klein grass; BG, Bermuda grass; BW, body weight; ADG, average daily gain; DMI, dry matter intake.

**Table 3:** Effect of C3 and C4 forages on apparent nutrient digestibility in Korean crossbred black goats (%).

Items	C3 Forages		C4 Forages		SEM	*p*-Value	
	RG	TG	KG	BG		(Forage Type)	(C3/C4 Pathways)
DM	65.4	64.8	63.8	62.4	0.63	0.358	0.134
CP	67.7	72.6	69.2	70.3	0.70	0.105	0.791
EE	76.8 ^a^	79.0 ^a^	64.8 ^b^	76.6 ^a^	1.96	0.007	0.011
NDF	51.2	47.7	48.5	44.8	1.03	0.113	0.115
ADF	45.7 ^a^	45.3 ^a^	41.7 ^ab^	33.3 ^b^	1.74	0.015	0.009
Ash	45.4	49.8	48.7	46.4	1.30	0.685	0.982
NFC ^1^	87.6	91.6	90.3	93.0	1.36	0.171	0.160

^1^ Non-fiber carbohydrate (%) = 100 − (CP + EE + NDF+ Ash) [24]. ^a,b^ Means with different superscripts in the same row are significantly different (*p* < 0.05). RG, Italian ryegrass; TG, timothy grass); KG, Klein grass; BG, Bermuda grass; DM, dry matter; CP, crude protein; N, nitrogen; EE, ether extract; NDF, neutral detergent fiber; ADF, acid detergent fiber; NFC, non-fiber carbohydrate.

**Table 4:** Nitrogen balance and utilization efficiency in Korean crossbred black goats fed different forage types.

Items	C3 Forages		C4 Forages		SEM	*p*-Value	
	RG	TG	KG	BG		(Forage Type)	(C3/C4 Pathways)
NI (g/d)	20.2 ^ab^	17.2 ^b^	22.3 ^a^	20.3 ^ab^	0.77	0.027	0.018
FN (g/d)	6.5	4.8	6.8	6.0	0.29	0.096	0.139
UN, (g/d)	4.0 ^ab^	3.7 ^ab^	7.1 ^a^	3.7 ^b^	0.75	0.025	0.074
DN (g/d)	13.7 ^ab^	12.4 ^b^	15.5 ^a^	14.2 ^ab^	0.52	0.021	0.008
ND (g/d)	67.7	72.5	69.3	70.3	0.01	0.105	0.791
TN (g/d)	10.5 ^ab^	7.4 ^b^	14.0 ^a^	9.7 ^b^	0.81	0.011	0.024
TN/NI (%)	53.5	43.4	63.4	49.7	4.32	0.144	0.332
RN (g/d)	9.7	9.8	8.3	10.6	0.99	0.261	0.529
RN/NI (%)	46.5	46.6	36.6	50.3	4.32	0.144	0.332
RN/DN (%)	68.1	78.0	52.7	71.6	6.02	0.152	0.368

^a,b^ Means with different superscripts in the same row are significantly different (*p* < 0.05). RG, Italian ryegrass; TG, timothy grass; KG, Klein grass; BG, Bermuda grass; NI, nitrogen intake; FN, fecal nitrogen; UN, urinary nitrogen; DN, digestible N (absorbed N); ND, N digestibility (N absorption); TN, total excreted N; TN/NI, total excreted N/nitrogen intake (N excretion); RN, retained N; RN/NI, retained N/nitrogen intake (N retention; utilization efficiency of N); RN/DN, retained N/digestible N (biological value of N).

## Data Availability

The original contributions of this study are incorporated within the article. For further information or requests regarding the data, please contact the corresponding author.

## Abbreviations

RG	Italian ryegrass
TG	Timothy grass
KG	Klein grass
BG	Bermuda grass
N	Nitrogen
PEP	Phosphoenolpyruvate
DM	Dry matter
NDF	Neutral detergent fiber
ADF	Acid detergent fiber
BW	Body weight
ADG	Average daily gain
CP	Crude protein
EE	Ether extract
DMI	Dry matter intake
FN	Fecal nitrogen
UN	Urinary nitrogen
DN	Digestible nitrogen
NI	Nitrogen intake
ND	Nitrogen digestibility
TN	Total excreted nitrogen
RN	Retained nitrogen
